# Tumor-originated exosomal lncUEGC1 as a circulating biomarker for early-stage gastric cancer

**DOI:** 10.1186/s12943-018-0834-9

**Published:** 2018-04-24

**Authors:** Ling-Yun Lin, Li Yang, Qiang Zeng, Lin Wang, Mao-Li Chen, Ze-Hang Zhao, Guo-Dong Ye, Qi-Cong Luo, Pei-Yu Lv, Qi-Wei Guo, Bo-An Li, Jian-Chun Cai, Wang-Yu Cai

**Affiliations:** 1 Department of Gastrointestinal Surgery, Zhongshan Hospital of Xiamen University, Xiamen, Fujian China; 2 Institute of Gastrointestinal Oncology, Medical College of Xiamen University, Xiamen, Fujian China; 3 Present Address: State Key Laboratory of Cellular Stress Biology, Innovation Center for Cell Signaling Network, School of Life Sciences, Xiamen University, Xiamen, Fujian China; 4 Xiamen LifeInt Technology Co., Ltd, Xiamen, Fujian China

**Keywords:** Exosome, lncRNA, Carcinoembryonic antigen, Early gastric cancer, Chronic atrophic gastritis, Diagnosis

## Abstract

Conventional tumor markers for non-invasive diagnosis of gastric cancer (GC) exhibit insufficient sensitivity and specificity to facilitate detection of early gastric cancer (EGC). We aimed to identify EGC-specific exosomal lncRNA biomarkers that are highly sensitive and stable for the non-invasive diagnosis of EGC. Hence, in the present study, exosomes from the plasma of five healthy individuals and ten stage I GC patients and from culture media of four human primary stomach epithelial cells and four gastric cancer cells (GCCs) were isolated. Exosomal RNA profiling was performed using RNA sequencing to identify EGC-specific exosomal lncRNAs. A total of 79 and 285 exosomal RNAs were expressed at significantly higher levels in stage I GC patients and GCCs, respectively, than that in normal controls. Through combinational analysis of the RNA sequencing results, we found two EGC-specific exosomal lncRNAs, lncUEGC1 and lncUEGC2, which were further confirmed to be remarkably up-regulated in exosomes derived from EGC patients and GCCs. Furthermore, stability testing demonstrates that almost all the plasma lncUEGC1 was encapsulated within exosomes and thus protected from RNase degradation. The diagnostic accuracy of exosomal lncUEGC1 was evaluated, and lncUEGC1 exhibited AUC values of 0.8760 and 0.8406 in discriminating EGC patients from healthy individuals and those with premalignant chronic atrophic gastritis, respectively, which was higher than the diagnostic accuracy of carcinoembryonic antigen. Consequently, exosomal lncUEGC1 may be promising in the development of highly sensitive, stable, and non-invasive biomarkers for EGC diagnosis.

**Electronic supplementary material:**

The online version of this article (10.1186/s12943-018-0834-9) contains supplementary material, which is available to authorized users.

## Main text

Gastric cancer (GC) is the fourth most common cancer and the second leading cause of cancer-related death worldwide, and it remains the most lethal cancer in Asia, including China [1]. GC patient survival rates can be improved by early diagnosis and treatment. Currently, EGC diagnosis involves invasive and non-invasive methods, which are painful or exhibit low sensitivity. Worse still, some gastric premalignant and precursor lesions, such as *Helicobacter pylori* (HP) infection, intestinal metaplasia (IM) and chronic atrophic gastritis (CAG), increase the difficulty of detecting EGC through existing non-invasive diagnostic methods [2].

Circulating RNAs in serum or plasma have emerged as novel non-invasive diagnostic biomarkers [3]. However, a large proportion of the circulating RNAs are easily affected by RNAs released by circulating dysfunctional cells. Previous studies have confirmed that cancerous cells secrete exosomes into the peripheral circulation, and exosomal RNAs can more accurately reflect changes in cancer cells during tumor progression [4]. In addition, exosomes can protect RNA from degradation by endogenous RNases, thereby enhancing the stability of exosomal RNAs in circulating blood [5]. Therefore, circulating exosomal RNAs, especially miRNAs, have emerged as promising biomarkers for the detection of early-stage cancers [6–8]; however, it remains uncertain whether tumor cell-originated exosomal lncRNAs in plasma can effectively diagnose early-stage cancer. Thus, in this study, we focused on lncRNAs in circulating exosomes that originated from cancer cells to determine their potential value for EGC diagnosis.

### RNA sequencing-based screening for identification of EGC-specific exosomal lncRNA

**Fig. 1:**
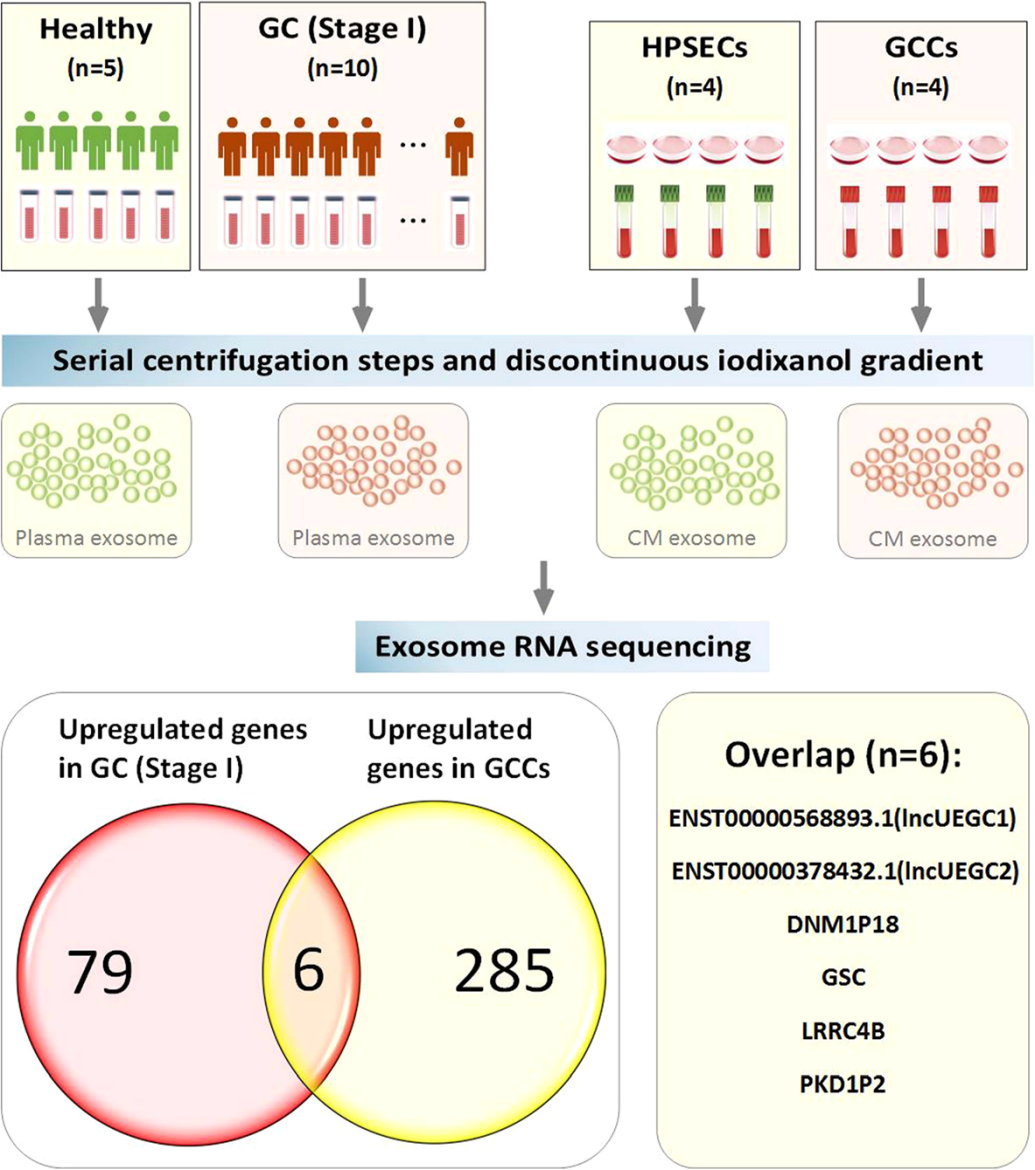
The screening model for identification of EGC-specific exosomal lncRNA. RNA sequencing-based screening was used to detect lncRNA and mRNA levels in exosomal fractions of plasma samples from patients with stage I GC and healthy controls as well as culture media (CM) from GCCs and HPSECs

The screening strategy for identification of EGC-specific exosomal lncRNAs is illustrated in Fig. 1. Plasma samples from patients diagnosed with stage I GC (*n* = 10) and healthy individuals (*n* = 5) (Additional file 1: Table S1) and culture media (CM) from different GCCs (AGS, KATO III, NCI-N87, and Hs 746 T; *n* = 4) and HPSECs (*n* = 4) were collected. Then, exosome-enriched fractions were prepared using step-wise centrifugation-ultracentrifugation and discontinuous iodixanol gradient methods. Transmission electron microscopy (TEM) and NanoSight particle tracking were used to characterize and quantify the isolated exosomes (Additional file 2: Figure S1A-D). Moreover, western blotting revealed the presence of specific exosome marker proteins (CD9 and CD63) and the absence of the negative control protein tubulin in the exosome-enriched fractions (Additional file 2: Figure S1E-F).

Next, exosomal RNA was purified and analyzed via RNA sequencing on an HiSeq3000 instrument. Based on the RNA sequencing data, 79 exosomal lncRNAs and mRNAs were up-regulated by more than 2-fold in stage I GC patients (*n* = 10) compared with healthy individuals (*n* = 5) (*Q* < 0.00001) (Additional file 2: Figure S1G, Additional file 3: Table S2). Previous studies have demonstrated that circulating exosomal lncRNA ZFAS1, LINC00152 and lncRNA HOTTIP levels were significantly elevated in GC patients compared with those in healthy controls [9–11]; however, these lncRNAs were not found in our up-regulated exosomal RNA profile, likely because they are not present in EGC. In our previous study, we could not exclude the possibility that these RNAs are secreted from other cells, including immune and inflammatory cells, which are coincidentally present at primary tissue lesions. Thus, exosomal RNA from the CM of four HPSECs and four GCCs was also analyzed via RNA sequencing. Two hundred eighty-five exosomal lncRNAs and mRNAs were up-regulated by more than 2-fold in culture medium from GCCs (*n* = 4) compared with HPSECs (*n* = 4) (*Q* < 0.00001) (Additional file 2: Figure S1H, Additional file 4: Table S3). We combined the results and found that six genes, ENST00000568893.1 (lncUEGC1), ENST00000378432.1 (lncUEGC2), DNM1P18, GSC, LRRC4B, and PKD1P2, were significantly up-regulated in both of the groups (Fig. 1). Hence, we focused on the two potential EGC-specific exosomal lncRNAs with the highest up-regulated fold changes and basic expression levels among the six genes and named them lncUEGC1 and lncUEGC2 (lncRNA up-regulated in the exosomes of gastric cancer).

### Verification of exosomal lncUEGC1 and lncUEGC2 up-regulation in EGC specimens and GCCs

**Fig. 2:**
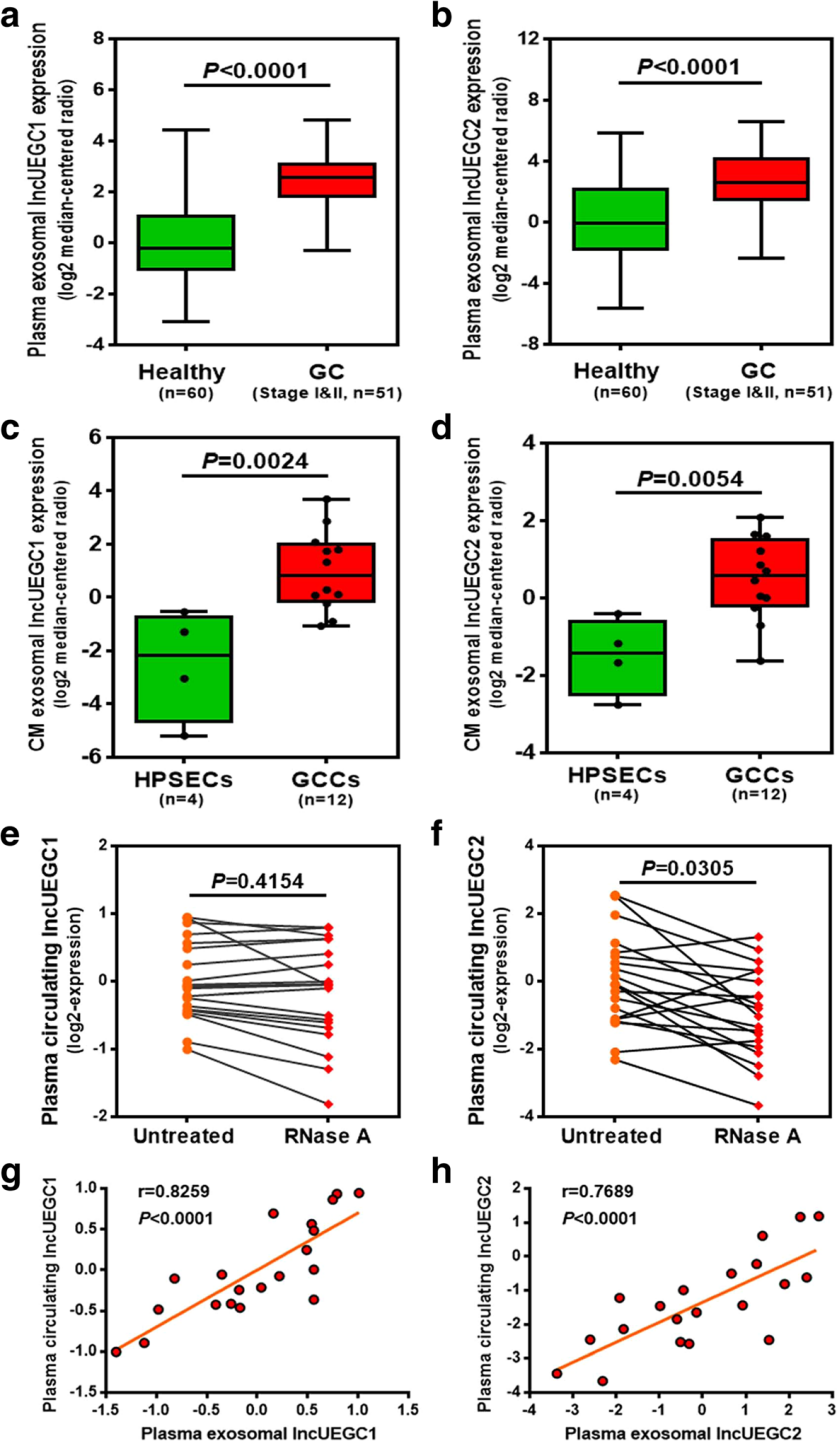
Expression pattern and stability testing of exosomal lncUEGC1 and lncUEGC2 in EGC specimens and GCCs. (**a** and **b**) qPCR analysis of the relative plasma exosomal lncUEGC1 (**a**) and lncUEGC2 (**b**) levels in stage I and II GC patients (*n* = 51) and healthy controls (*n* = 60). (**c** and **d**) qPCR analysis of the relative exosomal lncUEGC1 (**c**) and lncUEGC2 (**d**) levels in GCC (*n* = 12) and HPSEC (*n* = 4) culture media. (**e** and **f**) qPCR analysis of the relative plasma circulating lncUEGC1 (**e**) and lncUEGC2 (**f**) levels in stage I GC patient plasma samples (*n* = 20) treated with or without RNase (2 μg/ml) for 20 min. (**g** and **h**) Positive correlations between plasma exosomal and plasma circulating lncUEGC1 (**g**) and lncUEGC2 (**h**) expression in stage I GC patients (*n* = 20) were determined using Pearson’ s correlation test (r and *P* values are shown in the graphs). Differences with *P* < 0.05 were considered statistically significant

To further study the robustness of the RNA-seq data, quantitative PCR (qPCR) was performed to analyze exosomal lncUEGC1 and lncUEGC2 expression in EGC specimens from the validation set (Additional file 1: Table S1) and GCCs. As shown in Fig. 2a and b, the relative plasma exosomal lncUEGC1 and lncUEGC2 expression levels were both significantly up-regulated in stage I and II GC patients (*n* = 51) compared with healthy controls (*n* = 60) (fold change > 5, *P* < 0.0001). Moreover, culture medium from 12 different GCCs and four HPSECs obtained via primary culture from four different healthy donors were used to prepare exosome-enriched fractions. The qPCR analysis results revealed that exosomal lncUEGC1 and lncUEGC2 were significantly up-regulated in GCC (*n* = 12) culture medium compared with those in HPSEC culture medium (*n* = 4) (lncUEGC1, fold change = 11.27, *P* = 0.0024; lncUEGC1, fold change = 4.01, *P* = 0.0054) (Fig. 2c-d). Collectively, these results were consistent with the RNA-seq data and suggested that lncUEGC1 and lncUEGC2 may have potential as EGC-specific exosomal lncRNAs for early non-invasive GC diagnosis.

### The stability testing of circulating lncUEGC1 and lncUEGC2 in EGC specimens

Although identification of circulating RNA in plasma is an emerging field for non-invasive diagnostic applications, the majority of long RNA molecules in plasma exhibit poor stability. To examine the stability of lncUEGC1 and lncUEGC2 in plasma, twenty stage I GC specimen plasma samples were directly treated with RNase. The relative plasma levels of total circulating lncUEGC1 were not significantly different between plasma treated with or without RNase (*P* = 0.4154) (Fig. 2e). However, the relative plasma levels of total circulating lncUEGC2 were down-regulated by approximately two-fold in plasma under RNase treatment (*P* = 0.0305) (Fig. 2f). We also evaluated lncUEGC1 and lncUEGC2 levels in exosomes-depleted plasma supernatants after ultracentrifugation. The expression of lncUEGC1 were abundant in exosomes instead of exosomes-depleted plasma, but the expression of lncUEGC2 were not significantly different between exosomes and exosomes-depleted plasma (Additional file 5: Figure S2), which revealed that a proportion of lncUEGC2 was not encapsulated within exosomes.

Moreover, we analyzed the correlation between the relative lncUEGC1 and lncUEGC2 levels in plasma exosomal RNA and plasma total circulating RNA. The relative lncUEGC1 and lncUEGC2 levels were both closely related in plasma exosomal RNA and plasma total circulating RNA according to the calculated correlation coefficients (Fig. 2g-h). Taken together, the above results suggest that almost all the plasma lncUEGC1 was encapsulated within exosomes and thus protected from RNase degradation rather than circulating freely in the plasma.

### Expression pattern and diagnostic accuracy of CEA, lncUEGC1 and lncUEGC2 in the validation set

**Fig. 3:**
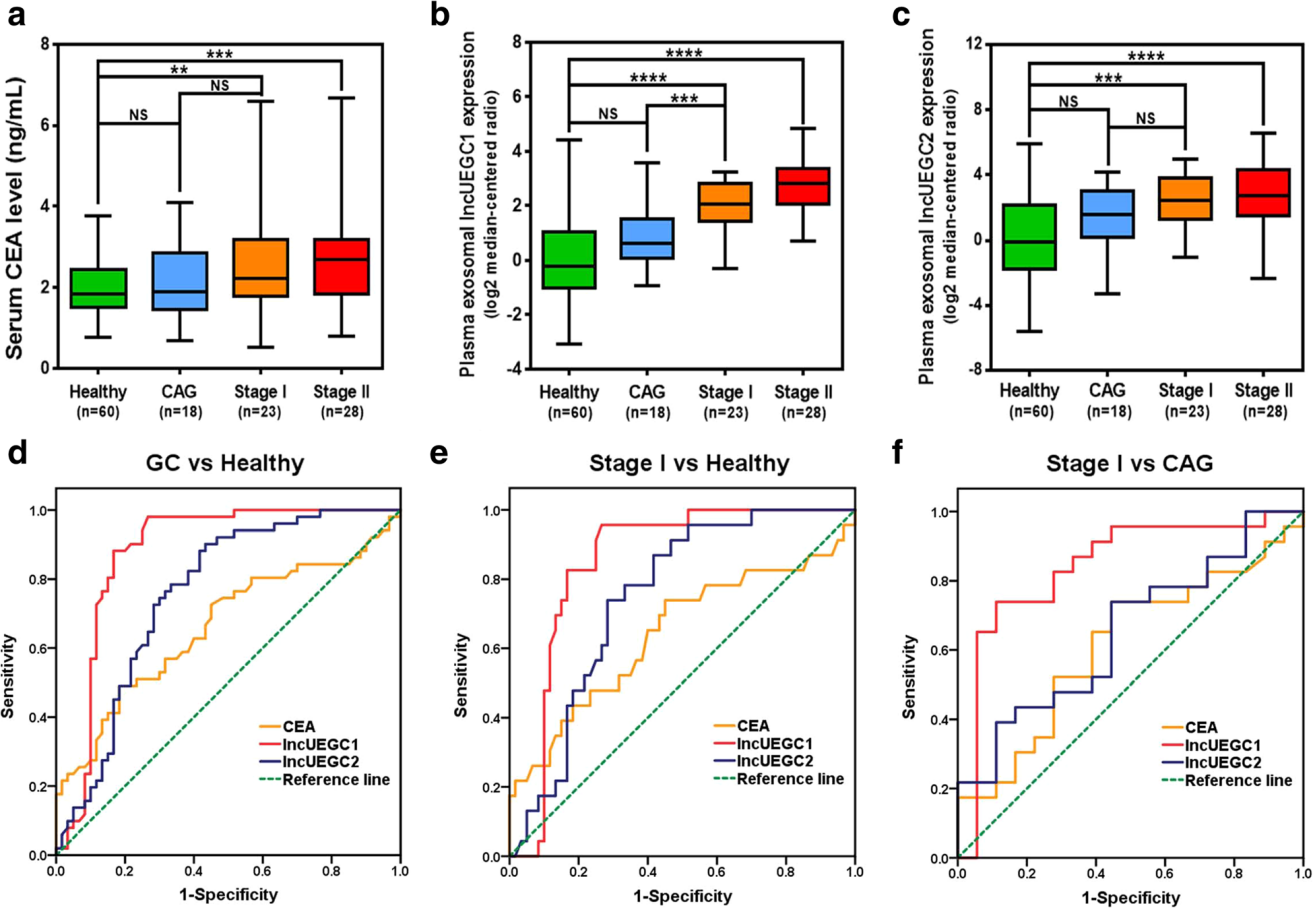
Expression pattern and diagnostic values of CEA, lncUEGC1 and lncUEGC2 in the validation set. **a** Serum CEA levels were detected in CAG (*n* = 18), stage I GC (*n* = 23), and stage II GC (*n* = 28) patients and in healthy controls (*n* = 60). **b** and **c** qPCR analysis of the relative plasma exosomal lncUEGC1 (**b**) and lncUEGC2 (**c**) levels in CAG (*n* = 18), stage I GC (*n* = 23), and stage II GC (*n* = 28) patients and in healthy controls (*n* = 60). **d** ROC curve of serum CEA levels, relative plasma exosomal lncUEGC1 and lncUEGC2 levels in stage I and II GC patients (*n* = 51) and healthy controls (*n* = 60). **e** ROC curve of serum CEA levels and relative plasma exosomal lncUEGC1 and lncUEGC2 levels in stage I GC patients (*n* = 23) and healthy controls (*n* = 60). **f** ROC curve of serum CEA levels and relative plasma exosomal lncUEGC1 and lncUEGC2 levels in stage I GC patients (*n* = 23) and CAG patients (*n* = 18). Differences with *P* < 0.05 were considered statistically significant. NS, not significant; **P < 0.01; ***P < 0.001; ****P < 0.0001

To determine the diagnostic power of exosomal lncUEGC1 and lncUEGC2 and the conventional tumor marker CEA for EGC detection, we examined plasma exosomal lncUEGC1 and lncUEGC2 expression levels and serum CEA levels for their capacity to distinguish EGC patients from those with premalignant CAG lesions as well as from healthy controls. As shown in Fig. 3a-c, the relative plasma exosomal lncUEGC1 and lncUEGC2 expression levels and serum CEA levels were all significantly up-regulated in stage I (*n* = 23) or II (*n* = 28) GC patients compared with healthy controls (*n* = 60), but only plasma exosomal lncUEGC1 relative expression levels were significantly up-regulated in stage I (*n* = 23) GC patients compared with CAG patients (*n* = 18).

Furthermore, the receiver operating characteristic (ROC) curve (AUC) was generated to evaluate the diagnostic accuracy. Plasma exosomal lncUEGC1 exhibited an AUC value of 0.8760 (*P* < 0.0001) in distinguishing stage I and II GC patients (*n* = 51) from healthy controls (*n* = 60), while plasma exosomal lncUEGC2 exhibited an AUC = 0.7582 (*P* < 0.0001) and serum CEA exhibited an AUC = 0.6614 (*P* = 0.0035) (Fig. 3d, Additional file 6: Table S4). The AUC value of exosomal lncUEGC1 was significantly higher than those of the combined serum tumor markers (CEA, CA19-9, CA72-4 and CA12-5) and potential plasma miRNA biomarkers identified in recent studies of EGC diagnosis [12, 13]. More importantly, our diagnostic accuracy study selectively restricted GC patients to those with stage I and II GC, excluding advanced stages, which indicated that lncUEGC1 may be more convincing for EGC diagnosis than markers identified in previous studies investigating diagnostic accuracy in GC patients at all stages [14–16]. Similar results were achieved in distinguishing stage I GC patients (*n* = 23) from healthy controls (*n* = 60); plasma exosomal lncUEGC1 still showed better diagnostic efficiency, with an AUC = 0.8500 (*P* < 0.0001), compared with plasma exosomal lncUEGC2, with an AUC = 0.7486 (*P* = 0.0005), and serum CEA, with an AUC = 0.6424 (*P* = 0.0456) (Fig. 3e, Additional file 6: Table S4). Because it is difficult to distinguish between early-stage gastric cancer and premalignant lesions using the current liquid biopsy index, we further generated the ROC curve of plasma exosomal lncUEGC1 and lncUEGC2 and serum CEA in distinguishing stage I GC patients (*n* = 23) from CAG patients (*n* = 18). As shown in Fig. 3f and Additional file 6: Table S4, plasma exosomal lncUEGC1 exhibited a high AUC value of 0.8406 (*P* = 0.0002) compared with plasma exosomal lncUEGC2, with an AUC = 0.6522 (*P* = 0.0980), and serum CEA, with an AUC = 0.6123 (*P* = 0.2219). Collectively, these results provide evidence that plasma exosomal lncUEGC1 may serve as a primary diagnostic GC biomarker. Moreover, this index might effectively distinguish between EGC and premalignant lesions. All methods and meterials used during this study are included in Additional file 7.

## Conclusions

Our research is the first use to use exosomal long chain RNA sequencing to systematically and thoroughly screen potential EGC biomarkers. The unique properties of exosomes, including their ability to embed cancer-originated RNAs, their stability in circulation and their reproducible detection, were revealed in this study. EGC-originated exosomal lncUEGC1 may be promising for the development of highly sensitive, stable, and non-invasive biomarkers for EGC diagnosis.

## Additional files

Additional file 1: **Table S1.** Clinical characteristics of patients and healthy individuals of all sets. (PDF 176 kb)

Additional file 2: **Figure S1.** Characterization and quantification of exosome levels and heatmap of the up-regulated exosomal lncRNAs and mRNAs identified in the screening. (A) Transmission electron microscopy (TEM) revealed the phenotype of exosomes isolated from stage I GC patient and healthy control plasma samples. Scale bar, 100 nm. (B) TEM revealed the phenotype of exosomes isolated from GCC and HPSEC culture media. Scale bar, 200 nm. (C and D) NanoSight particle tracking analysis of the size distributions and number of exosomes isolated from stage I GC patient and healthy control plasma samples (C) and isolated from GCC and HPSEC culture media (D). (E and F) Western blot showing the presence of specific exosome marker proteins (CD9 and CD63) and the absence of tubulin in exosomes derived from stage I GC patient and healthy control plasma samples (E) and derived from GCC and HPSEC culture media (F). (G) Heatmap of the 79 up-regulated exosomal lncRNAs and mRNAs between the plasma samples of stage I GC patients (*n* = 10) and healthy controls (*n* = 5). (H) Heatmap of the 285 up-regulated exosomal lncRNAs and mRNAs between GCC (*n* = 4) and HPSEC (*n* = 4) culture media. (PDF 395 kb)

Additional file 3: **Table S2.** Seventy-nine exosomal lncRNAs and mRNAs up-regulated by more than 2-fold in stage I GC patients (*n* = 10) compared with healthy individuals (*n* = 5) (Q < 0.00001). (XLSX 21 kb)

Additional file 4: **Table S3.** Two hundred eighty-five exosomal lncRNAs and mRNAs up-regulated by more than 2-fold in culture medium from GCCs (*n* = 4) compared with HPSECs (*n* = 4) (*Q < 0.00001*). (XLSX 47 kb)

Additional file 5: **Figure S2.** qPCR analysis of the relative lncUEGC1 and lncUEGC2 levels in exosomes and exosomes-depleted plasma from stage I and II GC patients (*n* = 5). Differences with *P* < 0.05 were considered statistically significant. (PDF 210 kb)

Additional file 6: **Table S4.** Performance of CEA, IncUEGC1 and IncUEGC2 levels in the classification of GC and Healthy subsets, Stage I and Healthy subsets, Stage I and CAG subsets. (PDF 154 kb)

Additional file 7: Methods. (PDF 289 kb)

## Abbreviations

CAG: Chronic atrophic gastritis
CEA: Carcinoembryonic antigen
CM: Culture media
EGC: Early gastric cancer
GC: Gastric cancer
GCCs: Gastric cancer cells
HP: *Helicobacter pylori*
HPSECs: Human primary stomach epithelial cells
IM: Intestinal metaplasia
lncRNAs: Long non-coding RNAs
lncUEGC: LncRNA up-regulated in the exosomes of gastric cancer
ROC: Receiver operating characteristic
TEM: Transmission electron microscopy
