# Supplementary material for: Tumor-originated exosomal lncUEGC1 as a circulating biomarker for early-stage gastric cancer
Source: Mol Cancer. 2018 Apr 24;17:84. doi: 10.1186/s12943-018-0834-9 (PMC5978993; doi:10.1186/s12943-018-0834-9)
Supplement: Supplementary file 1 — Table S1. Clinical characteristics of patients and healthy individuals of all sets. (PDF 176 kb) [file 12943_2018_834_MOESM1_ESM.pdf]

Table S1. Clinical characteristics of patients and healthy individuals of all sets.

Categories	Testing set (n=15)			Validation set (n=129)				
	Healthy (n=5)	GC (n=10)	*P*-value[a]	Healthy (n=60)	GC (n=51)	*P*-value[a]	CAG (n=18)	*P*-value[a]
Age(years)	57.2±6.8	59.4±10.8	0.687	58.3±8.6	61.1±9.8	0.108	59.1±6.4	0.708
Gender n(%)								
Male	4(80.0)	8(80.0)	1.000	38(63.3)	31(60.8)	0.846	13(72.2)	0.580
Female	1(20.0)	2(20.0)		22(36.7)	20(39.2)		5(27.8)	
Tumor stage n(%)								
Ia		8(80.0)			16(31.4)			
Ib		2(20.0)			7(13.7)			
IIa		0			15(29.4)			
IIb		0			13(25.5)			

Abbreviation: GC, gastric cancer; CAG, chronic atrophic gastritis;

[a]Compared to Healthy group.
