# Supplementary material for: Tumor-originated exosomal lncUEGC1 as a circulating biomarker for early-stage gastric cancer
Source: Mol Cancer. 2018 Apr 24;17:84. doi: 10.1186/s12943-018-0834-9 (PMC5978993; doi:10.1186/s12943-018-0834-9)
Supplement: Supplementary file 2 — Figure S1. Characterization and quantification of exosome levels and heatmap of the up-regulated exosomal lncRNAs and mRNAs identified in the screening. (A) Transmission electron microscopy (TEM) revealed the phenotype of exosomes isolated from stage I GC patient and healthy control plasma samples. Scale bar, 100 nm. (B) TEM revealed the phenotype of exosomes isolated from GCC and HPSEC culture media. Scale bar, 200 nm. (C and D) NanoSight particle tracking analysis of the size distributions and number of exosomes isolated from stage I GC patient and healthy control plasma samples (C) and isolated from GCC and HPSEC culture media (D). (E and F) Western blot showing the presence of specific exosome marker proteins (CD9 and CD63) and the absence of tubulin in exosomes derived from stage I GC patient and healthy control plasma samples (E) and derived from GCC and HPSEC culture media (F). (G) Heatmap of the 79 up-regulated exosomal lncRNAs and mRNAs between the plasma samples of stage I GC patients (n = 10) and healthy controls (n = 5). (H) Heatmap of the 285 up-regulated exosomal lncRNAs and mRNAs between GCC (n = 4) and HPSEC (n = 4) culture media. (PDF 395 kb) [file 12943_2018_834_MOESM2_ESM.pdf]

Figure S1

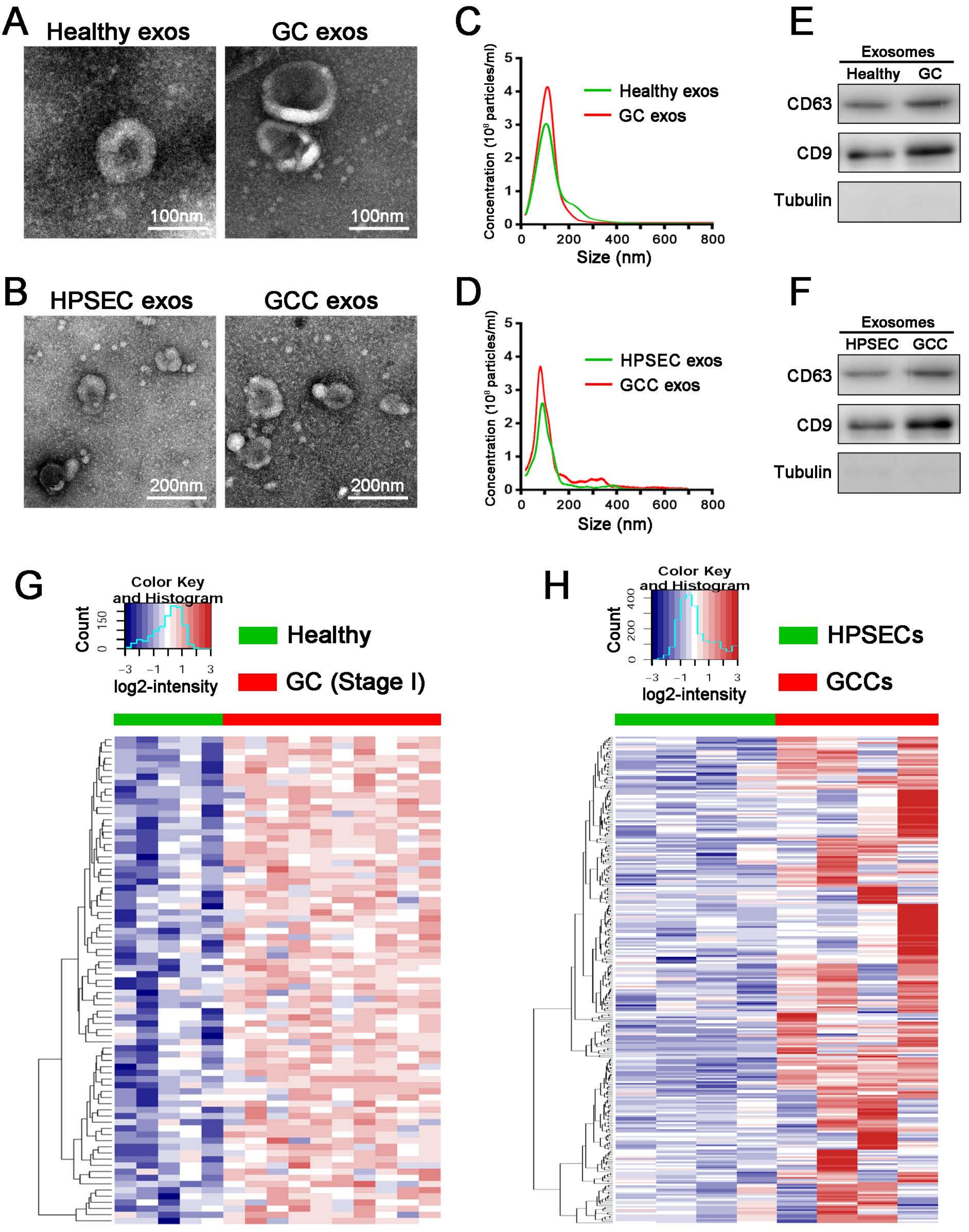
A
Healthy exos
GC exos
100nm
100nm
B
HPSEC exos
GCC exos
200nm
200nm
C
Concentration (10^8 particles/ml)
Size (nm)
Healthy exos
GC exos
D
Concentration (10^8 particles/ml)
Size (nm)
HPSEC exos
GCC exos
E
Exosomes
Healthy
GC
CD63
CD9
Tubulin
F
Exosomes
HPSEC
GCC
CD63
CD9
Tubulin
G
Color Key and Histogram
Count
log2-intensity
Healthy
GC (Stage I)
H
Color Key and Histogram
Count
log2-intensity
HPSECs
GCCs

Figure S1. Characterization and quantification of exosome levels and heatmap of the up-regulated exosomal lncRNAs and mRNAs identified in the screening. (A) Transmission electron microscopy (TEM) revealed the phenotype of exosomes isolated from stage I GC patient and healthy control plasma samples. Scale bar, 100 nm. (B) TEM revealed the phenotype of exosomes isolated from GCC and HPSEC culture media. Scale bar, 200 nm. (C and D) NanoSight particle tracking analysis of the size distributions and number of exosomes isolated from stage I GC patient and healthy control plasma samples (C) and isolated from GCC and HPSEC culture media (D). (E and F) Western blot showing the presence of specific exosome marker proteins (CD9 and CD63) and the absence of tubulin in exosomes derived from stage I GC patient and healthy control plasma samples (E) and derived from GCC and HPSEC culture media (F). (G) Heatmap of the 79 up-regulated exosomal lncRNAs and mRNAs between the plasma samples of stage I GC patients (n=10) and healthy controls (n=5). (H) Heatmap of the 285 up-regulated exosomal lncRNAs and mRNAs between GCC (n=4) and HPSEC (n=4) culture media.
