# Supplementary material for: Tumor-originated exosomal lncUEGC1 as a circulating biomarker for early-stage gastric cancer
Source: Mol Cancer. 2018 Apr 24;17:84. doi: 10.1186/s12943-018-0834-9 (PMC5978993; doi:10.1186/s12943-018-0834-9)
Supplement: Supplementary file 5 — Figure S2. qPCR analysis of the relative lncUEGC1 and lncUEGC2 levels in exosomes and exosomes-depleted plasma from stage I and II GC patients (n = 5). Differences with P < 0.05 were considered statistically significant. (PDF 210 kb) [file 12943_2018_834_MOESM5_ESM.pdf]

Figure S2

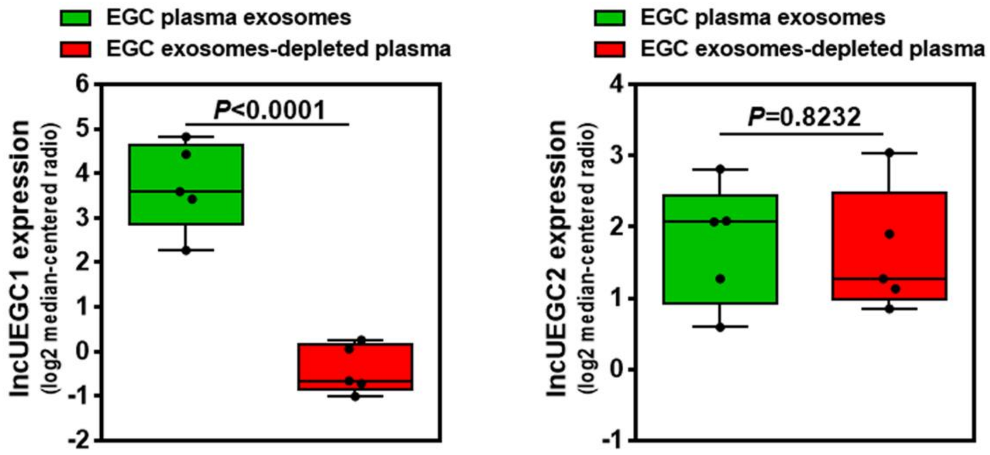

Figure S2. qPCR analysis of the relative lncUEGC1 and lncUEGC2 levels in exosomes and exosomes-depleted plasma from stage I and II GC patients (n=5). Differences with *P*<0.05 were considered statistically significant.
