# Supplementary material for: Tumor-originated exosomal lncUEGC1 as a circulating biomarker for early-stage gastric cancer
Source: Mol Cancer. 2018 Apr 24;17:84. doi: 10.1186/s12943-018-0834-9 (PMC5978993; doi:10.1186/s12943-018-0834-9)
Supplement: Supplementary file 6 — Table S4. Performance of CEA, IncUEGC1 and IncUEGC2 levels in the classification of GC and Healthy subsets, Stage I and Healthy subsets, Stage I and CAG subsets. (PDF 154 kb) [file 12943_2018_834_MOESM6_ESM.pdf]

Table S4. Performance of CEA, lncUEGC1 and lncUEGC2 levels in the classification of GC and Healthy subsets, Stage I and Healthy subsets, Stage I and CAG subsets.

Comparison	AUC [95% CI]	*P*-value	Sens	Spec	Acc
**GC vs Healthy**					
CEA	0.6614 [0.5574, 0.7655]	0.0035	49.02%	81.67%	66.67%
lncUEGC1	0.8760 [0.8035, 0.9484]	< 0.0001	88.24%	83.33%	85.59%
lncUEGC2	0.7582 [0.6678, 0.8485]	< 0.0001	90.20%	56.67%	72.07%
**Stage I vs Healthy**					
CEA	0.6424 [0.4969, 0.7879]	0.0456	73.91%	55.00%	60.24%
lncUEGC1	0.8500 [0.7650, 0.9350]	< 0.0001	95.65%	73.33%	79.52%
lncUEGC2	0.7486 [0.6438, 0.8533]	0.0005	73.91%	71.67%	72.29%
**Stage I vs CAG**					
CEA	0.6123 [0.4372, 0.7874]	0.2219	73.91%	55.56%	65.85%
lncUEGC1	0.8406 [0.7064, 0.9747]	0.0002	73.91%	88.89%	80.49%
lncUEGC2	0.6522 [0.4836, 0.8207]	0.0980	73.91%	55.56%	65.85%

Abbreviation: CI, confidence interval; Sens, sensitivity; Spec, specificity; Acc, overall accuracy.
