# Supplementary material for: Tumor-originated exosomal lncUEGC1 as a circulating biomarker for early-stage gastric cancer
Source: Mol Cancer. 2018 Apr 24;17:84. doi: 10.1186/s12943-018-0834-9 (PMC5978993; doi:10.1186/s12943-018-0834-9)
Supplement: Supplementary file 7 — Methods. (PDF 289 kb) [file 12943_2018_834_MOESM7_ESM.pdf]

Methods

Cell culture

The human GC cell lines AGS, SNU-1, SNU-5, SNU-16, KATO III, NCI-N87, and Hs 746T were obtained from the American Type Culture Collection (ATCC, MD, USA), and the human GC cell lines MKN-28, MKN-45, HGC-27, BGC-823, and MGC80-3 were purchased from the Cell Bank of Type Culture Collection of the Chinese Academy of Sciences (Shanghai, China; http://www.cellbank.org.cn). Human primary stomach epithelial cells (HPSEC1, HPSEC2, HPSEC3, and HPSEC4) were purchased from the Cell Biologics Company (Chicago, USA; http://www.cellbiologics.net). Short tandem repeat sequencing was performed for human GC cell line authentication. Immunofluorescence staining of cytokeratin-18 and 19 and vimentin was used for HPSEC authentication. All cells used in this study were validated within 6 months of the experiments. PCR tests for mycoplasma were negative. Human GC cell lines were maintained in RPMI-1640 medium (Gibco, USA) containing 10% fetal bovine serum (Gibco, Grand Island, NY, USA) and 1% penicillin/streptomycin (Invitrogen, Carlsbad, CA, USA). HPSECs were cultured in Complete Epithelial Cell Medium (Cell Biologics, cat. no. M6621). All cells were grown at 37°C with 5% carbon dioxide.

Patients and clinical samples

Between January 2014 and October 2017, ten stage I GC patients were enrolled for testing studies and 51 EGC and 18 CAG patients were enrolled for validation studies; in addition, 5 and 60 healthy individuals matched for sex and age were included in the testing and validation sets, respectively. All patients were newly diagnosed with early-stage GC (stage I, II) or CAG, which was confirmed by two pathologists after surgery or endoscopy. Patients treated with chemotherapy or radiotherapy before blood collection were excluded. Tumors were staged according to the International Union against Cancer (UICC) guidelines. The detailed clinical characteristics of the patients are provided in Additional file 1: Table S1.

The blood samples included in this study were collected in vacuum blood tubes with

EDTA anticoagulant before surgery and pharmacotherapy and handled within 1 h after collection. Blood samples were subjected to centrifugation at 5000 rpm for 10 min at 4°C. The plasma was then stored at −80°C. Written informed consent was obtained from the participants before sampling. The studies were conducted in accordance with the International Ethical Guidelines for Biomedical Research Involving Human Subjects (CIOMS), and the research protocols were approved by the Clinical Research Ethics Committee of Zhongshan Hospital of Xiamen University.

Exosome isolation

For exosome collection from cell culture medium, cells plated in 15-cm dishes were maintained in 30 ml of RPMI-1640 medium (Gibco) supplemented with 10% Exo-FBS™ Exosome-depleted FBS (System Biosciences, CA, USA) and 1% penicillin/streptomycin (Invitrogen) for 48 h.

For exosome isolation and purification, 20 ml of plasma (1:10 diluted in phosphate-buffered saline (PBS)) or 20 ml of culture medium was pre-cleared using a 0.22-μm membrane filter (Millipore, USA) and concentrated by centrifugation at 110,000×g for 2 h at 4°C. The pellets containing total vesicles were resuspended in 100 μl of PBS and then processed using a discontinuous iodixanol (Thermo, USA) gradient. The gradient was formed by adding 1.2 ml of 40% iodixanol solution followed by careful layering of 1.2 ml each of 20%, 10% and 5% iodixanol solutions. Centrifugation was performed at 110,000×g for 2 h at 4°C. Twelve fractions with equal volumes (400 μl) were collected from the top of the gradient, and exosomes in fractions 6–9 (1:10 diluted in PBS) from the iodixanol gradient were pelleted by ultracentrifugation at 110,000×g for 2 h at 4°C. The pelleted exosomes were resuspended in 100 μl of PBS for subsequent analysis.

Exosome characterization and quantification

Exosomes were examined via transmission electron microscopy (TEM) with negative staining and quantified using a NanoSight NS300 instrument (Malvern Instruments Ltd. UK) equipped with NTA 3.0 analytical software (Malvern Instruments

Ltd. UK).

For immunoblotting, protein was extracted from an equal number of exosomes, suspended in SDS lysis buffer with proteinase inhibitors and quantified with bicinchoninic acid assays (Pierce, USA). Then, 20 μg of exosomal protein was resolved on a 12% SDS-PAGE gel, transferred to a PVDF membrane (Millipore, USA), and then incubated with the primary antibodies anti-CD63 (Immunoway, USA, cat. no. YT5525, 1:1000), anti-CD9 (Santa Cruz Biotechnology, USA, cat. no. sc-13118, 1:1000) and anti-tubulin (Abcam, UK, cat. no. ab7291, 1:5000) at 4°C overnight, followed by visualization using an Azure C300 Darkroom Eliminator (Azure Biosystems, USA).

RNA isolation from exosomes and plasma

Total exosomal RNA was isolated using an RNeasy Mini Kit (Qiagen, Hilden, Germany) according to the manufacturer's protocol. The HiPure Serum/Plasma miRNA Kit (Magen, China) was used for circulating plasma RNA isolation following the standard protocol provided by the manufacturer. RNA purity and concentration were assessed using a Qubit® 2.0 fluorometer and the Qubit RNA HS Assay Kit (Life Technologies, USA). RNA integrity was evaluated using an Agilent 2200 Tape Station (Agilent Technologies, USA).

Exosomal RNA sequencing

Exosomal RNA sequencing were performed by RiboBio Co. Ltd (Guangzhou, China; http://www.ribobio.com/). Exosomal total RNA was subjected to first strand and second strand cDNA synthesis followed by adaptor ligation and enrichment with a low cycle according to instructions for the NEBNext® Ultra™ Directional RNA Library Prep Kit for Illumina (NEB, USA).The purified library products were evaluated using an Agilent 2200 Tape Station (Agilent Technologies) and a Qubit® 2.0 Fluorometer (Life Technologies) and then diluted to 10 pM for in situ cluster generation on a HiSeq3000 paired-end flow cell followed by sequencing (2×150 bp) on the HiSeq3000 system. To highlight expression differences between groups, Audics was used to correct the *P*-

value and obtain the *Q*-value. A *Q*-value <0.00001 was considered extremely significant.

lncRNA quantification

cDNA was prepared from 30 ng of exosomal total RNA or 500 ng of plasma circulating RNA using a HiFi-MMLV cDNA kit from CWBIO (Beijing CoWin Biotech, Beijing, China, cat. no. CW0744). Quantitative PCR was performed using a CFX96 Touch Real-Time PCR detection system (Bio-Rad, CA, USA) with UltraSYBR Mixture from CWBIO, cat. no. CW0956. The lncRNA levels were normalized against a synthesized exogenous reference, λ polyA^{+} RNA (Takara, China), which was spiked in to normalize the exosomal total RNA or plasma circulating RNA levels at the onset of RNA reverse transcription according to the manufacturer's protocol provided with the External Standard Kit (λ polyA) for qPCR (Takara, China, cat. no. 3789). The expression levels were calculated using the $2^{-\Delta\Delta Ct}$ (where Ct is threshold cycle) method. The following primer sequences were used for quantitative PCR:

lncUEGC1: 5'- AGTGGCTTTTCTGGCTTTGC -3' (forward),
5'- TGGTGTCGCAGGCTCTAGGT -3' (reverse);

lncUEGC2: 5'- GCCCACGGCTTCAACCTG -3' (forward),
5'- TTAAACACACTCCCTACACTCGC -3' (reverse).

CEA determination

The serum CEA levels were measured using an Elecsys-electrochemical immune assay (Roche, USA) and were detected in a cobas 8000 modular analyzer with a cobas e 602 module (Roche, USA).

Statistical analysis

Statistical analyses were performed and graphs were constructed with GraphPad Prism 6.0 and SPSS 17.0 statistical software. The data are presented as the mean ± SD. Significant differences between the mean values of two groups were determined using Student's t-test. Pearson correlation analysis was performed to determine

correlations between two variables. Pearson’s chi-square test was used to analyze clinical variables. Clinicopathological diagnoses were used as the gold standard to assess diagnostic accuracy using receiver operating characteristic (ROC) curves generated with SPSS 17.0 software. All diagnostic metrics (i.e., sensitivity, specificity, and accuracy) were calculated using standard formulas. A *P*-value <0.05 was considered significant.
